# Silica–Resorcinol–Melamine–Formaldehyde Composite Aerogels as High-Performance Thermal Insulators

**DOI:** 10.1021/acsomega.1c04462

**Published:** 2022-04-21

**Authors:** Romain Civioc, Wim J. Malfait, Marco Lattuada, Matthias M. Koebel, Sandra Galmarini

**Affiliations:** † Laboratory for Building Energy Materials and Components, Swiss Federal Laboratories for Materials Science and Technology, Empa, 8600 Duebendorf, Switzerland; ‡ Department of Chemistry, University of Fribourg, Chemin du Musée 9, 1700 Fribourg, Switzerland

## Abstract

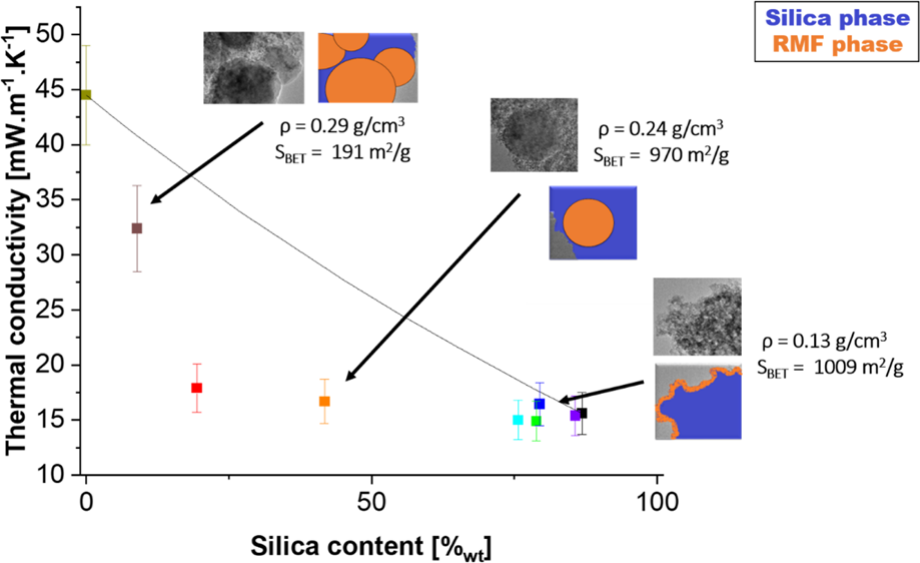

Here, we report the gelation and supercritical drying of ethanol-based silica–resorcinol–melamine–formaldehyde (RMF) composite aerogels with relative concentrations of initial reagents ranging from neat silica to neat RMF alcogels. The as-prepared materials are subsequently supercritically dried with carbon dioxide. Their properties include a thermal conductivity in the 15–20 mW·m^–1^·K^–1^ range even with a silica content as low as 20%_wt_. The possible reasons behind this interesting insulation performance and the mechanisms leading to the underlying gel structure are discussed in depth. A focus is made on the different gelation modes happening between the RMF and silica phases, from a coating of silica surfaces with RMF species to discontinuous RMF particles within a silica backbone and a continuous RMF backbone with isolated silica particles. The implications in terms of mechanical properties and thermal conductivity are elaborated upon. The initial ratio of silica–RMF species in this ethanol-based synthesis affects the micro- and macrostructure of the composites, resulting in materials with drastically different pore structures and thus an interesting array of possibilities for a new class of silica-organic composite aerogels, based on a sol–gel process.

## Introduction

Aerogels are a class of materials sometimes described as a new state of matter because their properties differ from those of equivalent bulk materials due to their nanostructured backbone and porosity and their high porosity (up to and above 99%, typically around 95%). Aerogels were first reported by Kistler in 1932^1^ and are still being studied extensively nowadays.^2,3^ Aside from silica aerogels, the pioneering and most commonly explored chemistry for those materials, other chemical systems have since then been studied as aerogel candidates, including metals^4−7^ and metal oxides, polymers^8−10^ and biopolymers,^11^ and carbon-based materials^12−14^ and their hybrids. The motivation behind exploring these different chemistries for aerogels is about elaborating interesting and innovating microstructures, chemistries, properties, and applications. The variety of their chemistry allows aerogels to be promising candidates for applications in thermal insulation,^15^ catalysis and gas separation,^16^ oil spillage absorption,^17^ and so forth.^8,14,18−20^

Resorcinol–formaldehyde resin (RF) is a particularly interesting candidate material and was first reported as an aerogel in a seminal paper by Pekala in 1989.^21^ Further down the road, these porous materials have been explored in detail^22−34^ and optionally can be doped with heteroatoms like nitrogen, for example, through the addition of urea^35−37^ or melamine,^22,23,38−40^ that is, effectively becoming resorcinol–melamine–formaldehyde (RMF) resins. Both silica and RF resins can be colloidal with a shared solvent system; for example, both can be synthesized in ethanol–water mixtures,^41^ which points toward a potential compatibility of the systems, yet there are only a few examples of their composite aerogel-related materials in the published literature.^42−48^ We hypothesize that an aerogel composite of silica and RMF can provide an interesting compromise between acceptable mechanical properties (one of the biggest drawbacks of silica aerogels) and a retention of silica aerogels’ exceptional thermal insulation properties. Indeed, their unique mesoporous microstructure^49^ allows them, through the Knudsen effect, to reach an extremely low thermal conductivity (λ = 12–15 mW·m^–1^·K^–1^ STP)—about half of that of standing air (λ ≈ 26 mW·m^–1^·K^–1^ STP).

In this work, we explore a silica–RMF composite aerogel system with different proportions of each precursor. Synthesis conditions were fixed except for the silica–RMF ratio, allowing for a direct comparison of the effect of chemical composition on the microstructure and bulk properties for a wide array of compositions. The hybrid aerogels are then scouted for performance by measuring their compressive strength and thermal conductivity under ambient conditions. The expected *versus* observed properties are discussed, and unexpected results and their possible causes are explored in detail.

## Materials and Methods

### Materials

Tetraethylorthosilicate (TEOS) was purchased from Evonik (Germany), and formaldehyde (ACS reagent, 37%_wt_ aqueous solution stabilized with 10–15%_wt_ methanol), resorcinol (ReagentPlus 99%), and melamine (99% purity) were all purchased from Sigma-Aldrich and used as-received without further purification. Ammonium hydroxide (ACS reagent 28.0–30.0% NH_3_ basis) was also purchased from Sigma-Aldrich and diluted to 5.5 mol·L^–1^ in deionized water before use. Absolute ethanol denatured with 5% isopropanol was used as the primary solvent for the synthesis.

### Synthesis

**Scheme 1:**
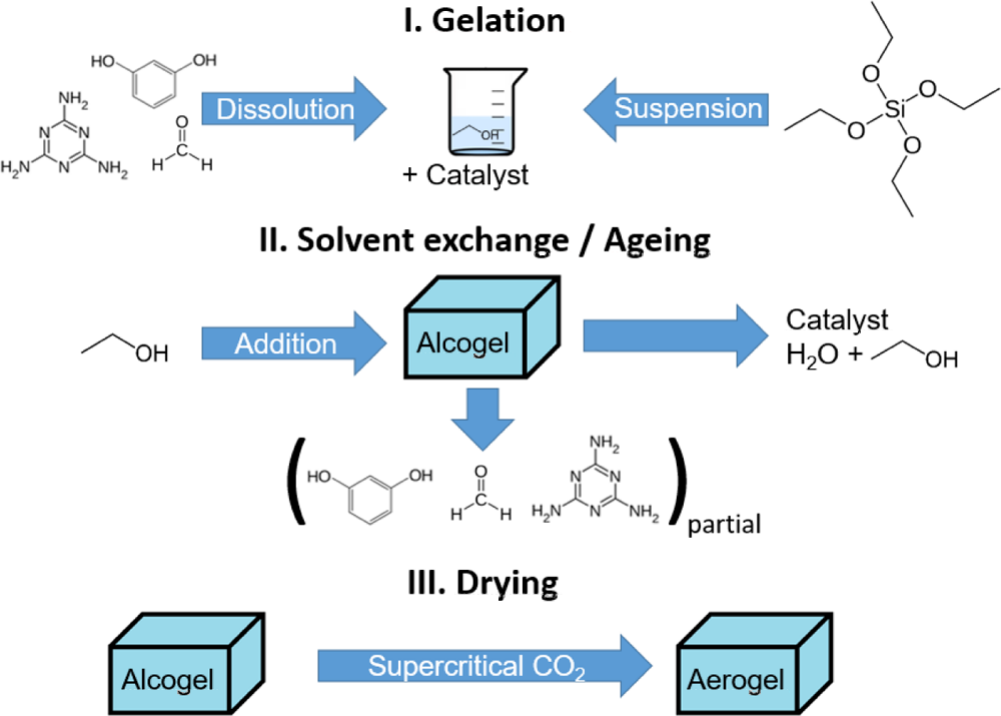
Synthesis Scheme: (I) Gelation Is Induced by the Addition of Catalytic Ammonia to the Mixed RMF and Silica Sol; (II) Water and Unreacted Monomers are Removed during Solvent exchange; and (III) Alcogel Is Converted Into an Aerogel Using Supercritical CO_2_ Drying

The composite aerogels were prepared by mixing a silica sol with an RMF sol (Scheme 1). The silica sol was produced according to a protocol from Pajonk *et al.* Shortly, TEOS is partially hydrolyzed by a controlled acidic environment at an elevated temperature, and the resulting monodisperse sol, with a 20%_wt_ SiO_2_ equivalent,^150^ is stored at 5 °C until usage. The preparation of the RMF ethanolic precursor sol, with a known solid-to-liquid ratio (0.379 g·mL^–1^), is described in detail in our previous publication.^41^ Briefly, melamine is dissolved in a water–ethanol–formaldehyde solution at 55 °C, and resorcinol is dissolved in ethanol also at 55 °C. The two sols are brought together and catalyzed by the addition of ammonia. For each experiment, the RMF and silica sols are mixed in pre-determined ratios. The ratio between the two precursor sols was selected such that the nominal silica content (eq 1) varied from 0 to 100%_nom_ SiO_2_ (Table 1).
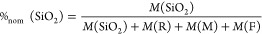
1
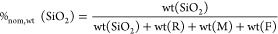
2

**Table 1:** Synthesis Conditions Used for the Preparation of the Different Silica–RMF Alcogels, Prior to Gelation^a^

name	S100	S90	S75	S63	S52	S31	S10	S05	RMF
SiO_2_ %_nom_	100	90.0	74.8	63.3	52.1	30.9	10.0	5.0	0
SiO_2_ %_nom,wt_	100	90.4	75.6	64.3	53.2	31.9	9.6	4.8	0
P750 sol (mL)	40.0	39.4	38.2	37.0	35.4	25.2	17.7	8.0	0
RMF sol (mL)	0	0.6	1.8	3.0	4.6	9.6	22.3	32.0	40.0

a The second line represents the molar percentage of silica species over silica plus RMF species, while the third line represents the same ratio in weight percentage.

After 15 min of stirring, ammonium hydroxide was added as a catalyst to reach a final ammonia concentration in the mixed sol of 0.128 mmol·L^–1^. The sols were then poured into closed polystyrene boxes in a 65 °C oven and left to age for 3 days. Solvents were exchanged daily with ethanol for a total of four washing steps over a 48 h period. The alcogels were then solvent-exchanged with liquid CO_2_ in an autoclave. The temperature and pressure were then increased to supercritical conditions (120 bars, 50 °C) for 5 h, followed by the release of CO_2_ at 50 °C.

### Characterization

#### Envelope Density

Envelope density measurements were carried out on a powder displacement device (GeoPyc 1360, Micromeritics) with a consolidation force of 4.0 N. On each sample, 10 consecutive measurements were realized, and the average value was retained. Measurements were repeated on samples from 5 different monoliths of each composition, for a total of 50 measurements. Given the low intrinsic error on this measurement with the type of material under investigation, the reported variation is the calculated standard deviation.

#### Skeletal Density

Skeletal density was measured by helium pycnometry (AccuPyc II 1340, Micromeritics) using a pressure of 134.447 kPa for purging and measurement cycles. Fifty purges followed by 20 measurements were realized on each sample, and the average value was retained. Measurements were repeated on samples from 5 different monoliths for each composition, for a total of 100 measurements. The reported deviation is the calculated standard deviation for these measurements.

#### Nitrogen Sorption

Nitrogen sorption isotherms were recorded at 77.4 K (3Flex, Micromeritics). Before each measurement, samples were degassed at 105 °C for 20 h at a pressure of 1.3 × 10^–2^ mbar. Accessible pore volumes and surface areas were measured using both classical analytical models (respectively Barrett–Joyner–Halenda^50^ between 2 and 50 nm and Brunauer–Emmett–Teller^51^ with the modified Rouquerol equation) and state-of-the-art nonlocal density functional theory (NLDFT) kernels, as recommended by the ISO-15901-3 standard.^52^ For NLDFT calculations, a cylindrical geometry was assumed for mesopores, based on Tarazona’s work.^53,54^

#### Thermogravimetric Analysis

Thermogravimetric analysis (TGA) curves were recorded under a reconstituted air atmosphere (20% O_2_ and 80% N_2_) with a heating rate of 10 K·min^–1^ (TG 209 F1, Netzsch).

#### Fourier-Transform Infrared Spectroscopy

Fourier-transform infrared spectroscopy (FTIR) was conducted on a Bruker spectrometer in attenuated total reflectance (ATR), using a diamond crystal, for wavenumbers ranging between 500 and 4000 cm^–1^. The spectra were normalized to the maximum intensity.

#### Elemental Analysis

Elemental analysis (C, H, and N) was carried out on a LECO TruSpec Micro. Before analysis, samples were dried in nitrogen sorption tubes overnight at 105 °C and 1.3 × 10^–2^ mbar. The C and H contents were determined by infrared spectroscopy. The N content was determined by measuring the thermal conductivity of the gas phase. The remaining weight was attributed to Si and O contents, assuming the absence of quantitative impurities. Measurements were carried out with an accuracy of ±0.3%.

#### Thermal Conductivity

Thermal conductivity was measured on a homemade guarded hot plate device^55^ with monolithic samples. Measurements were carried at least three times per composition with a relative error of 12%.

#### Mechanical Properties

Mechanical properties were evaluated through uniaxial compression tests performed on cylindrical samples preemptively flattened on both ends by using sandpaper, using a universal materials testing machine (Zwick/Z010, Zwick/Roell, Germany) equipped with a 2 kN force transducer cell (KAP-S, AST Gruppe GmbH, Germany). The compression rate was set to 1 mm/min, and the stress/strain response was measured until mechanical failure of the samples. The elastic moduli were calculated from the slope of the initial linear phase (3–5% strain) of the compression curve.

#### Solid-State NMR

Solid-state nuclear magnetic resonance (NMR) spectra were acquired on a Bruker AVANCE III system using a wide-bore 9.4 T magnet, corresponding to Larmor frequencies of 400.2 MHz for ^1^H, 100.6 MHz for ^13^C, and 79.5 MHz for ^29^Si with 7 mm-diameter zirconia rotors and at a magic angle spinning (MAS) rate of 4 kHz. ^1^H–^29^Si and ^1^H–^13^C cross polarization MAS NMR spectra were acquired to boost the sensitivity, with relatively long contact times (5 and 2 ms, respectively) to minimize the dependency of the spectral intensities on the ^1^H–X distance, but the spectra can only be considered in a semi-quantitative approach at best.

#### Transmission Electron Microscopy

Transmission electron microscopy (TEM) pictures were acquired on a JEM2200FS JEOL microscope at an operation voltage of 200 kV.

#### Macroscopic Colorimetry

Macroscopic colorimetry of samples was assessed on a Pantone uncoated colorimetric scale.^56^ Pictures were taken with a smartphone in a room with neutral white light.

## Results and Discussion

### Gelation times and Visual Appearance

Mixing two chemical systems requires finding compatible conditions for them to interact. Silica sols and RMF resins^41^ can both form wet gels in ethanol when catalyzed by ammonia. By finding the optimum NH_3_ catalyst concentration, composite silica–RMF alcogels of homogeneous appearance were obtained for nominal silica precursor contents spanning the entire range from 0 to 100%_nom_ (Table 1). Gelation time was dependent on relative concentrations, with longer gelation times for sols sporting a higher RMF content: from a few minutes for the neat silica sol, to a few hours for the neat RMF sol at 65 °C. After gelation, the gels underwent isotropic shrinkage, and a syneresis liquid could be observed for high silica precursor contents (≥52%_nom_), while gels with a high nominal RMF content (≤48%_nom_ SiO_2_) did not shrink right after gelation—they behaved like RMF alcogels in this respect. The color of the exchanged solvent was highly dependent on the composition, ranging from transparent to deep orange with discernible particles, indicating that reactants were partially washed out (see the Supporting Information)—leading to potential differences between the nominal concentration of the reactants and the final aerogel composition. The quantification of the solid mass in the exchange solvent by measuring changes in solvent density was unsuccessful as calculated mass losses were within the error range of the measurement.

**Figure 1:**
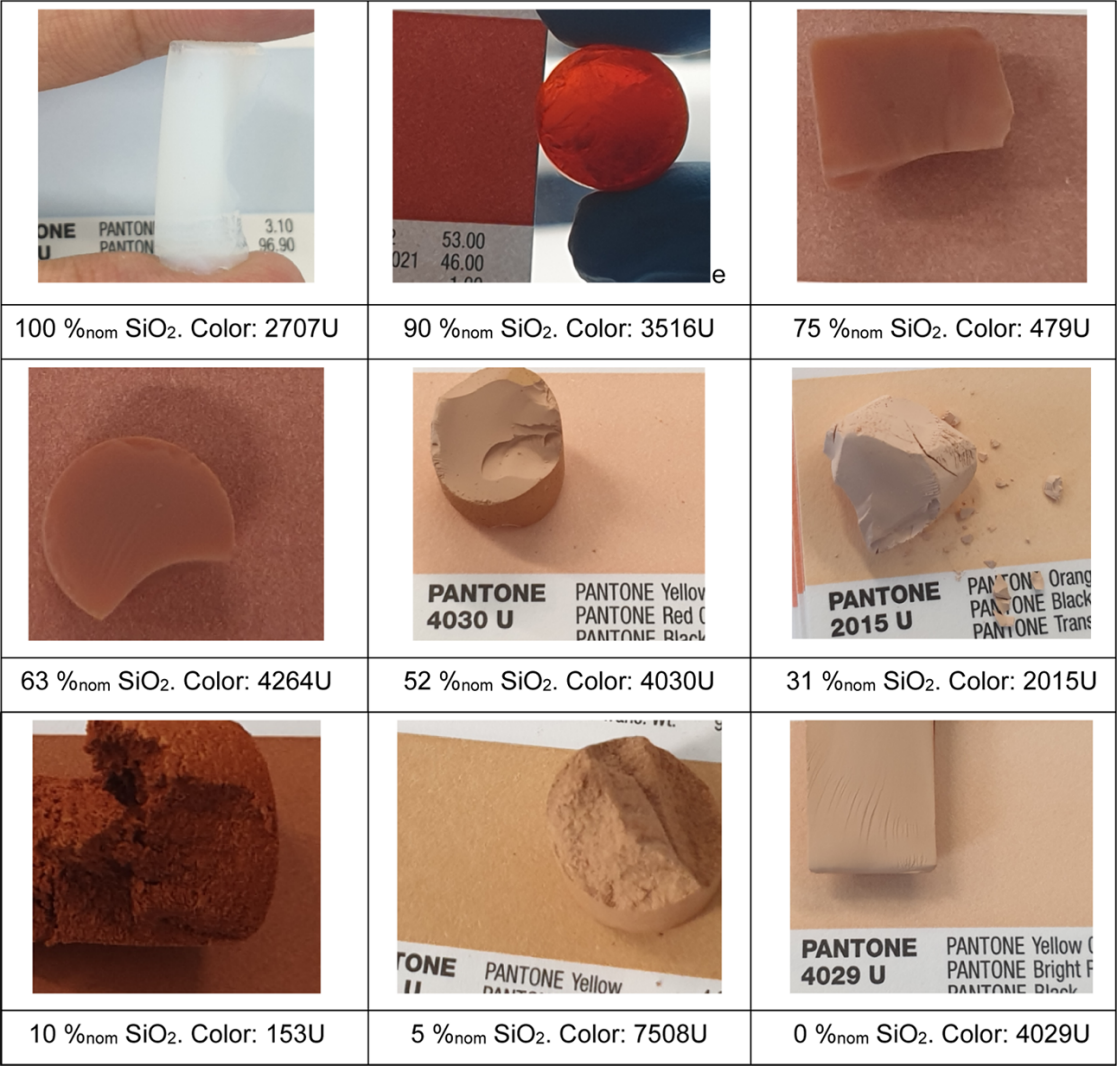
Visual appearance and the Pantone color code for silica–RMF composite aerogels, ranging from pure silica (100%_nom_ SiO_2_.) to pure RMF (0%_nom_ SiO_2_).

The following observations were made for the wet gels: samples with at least 31%_nom_ SiO_2_ had a visible shine and a smooth, quickly drying surface, as is also observed for pure silica gels, while samples with 10%_nom_ SiO_2_ or less showed a mat surface with no visible surface drying being observed, similar to the behavior of pure RMF resins. The corresponding aerogels, obtained after supercritical drying, varied greatly in physical appearance (Figure 1) and handling properties. For high silica nominal contents (≥90%_nom_ SiO_2_), the aerogels retain the typical optical translucency associated with low-density silica aerogels, indicating no significant widening of the microstructure nor the presence of large particles that would lead to optical diffraction. The red color of the 90%_nom_ SiO_2_ aerogel is strikingly different than that of the pure silica aerogel and also more intense than for the aerogels with a higher RMF content, despite its very low RMF content (see also below). We hypothesize that the strong coloration results from the high color strength of RMF compared to the nearly completely colorless silica, and the long optical path due to the high transparency compared to aerogels with a higher RMF content. As the nominal RMF content increases, the aerogels become increasingly less translucent (75 to 63%_nom_ SiO_2_) and then opaque—thus indicating increased light scattering, pointing toward the presence of larger microstructural features (pores or solid particles). The gradual shift in coloration, regardless of the degree of light scattering, is more indicative of changes of the chemical identity.

### Chemical Identity

**Figure 2:**
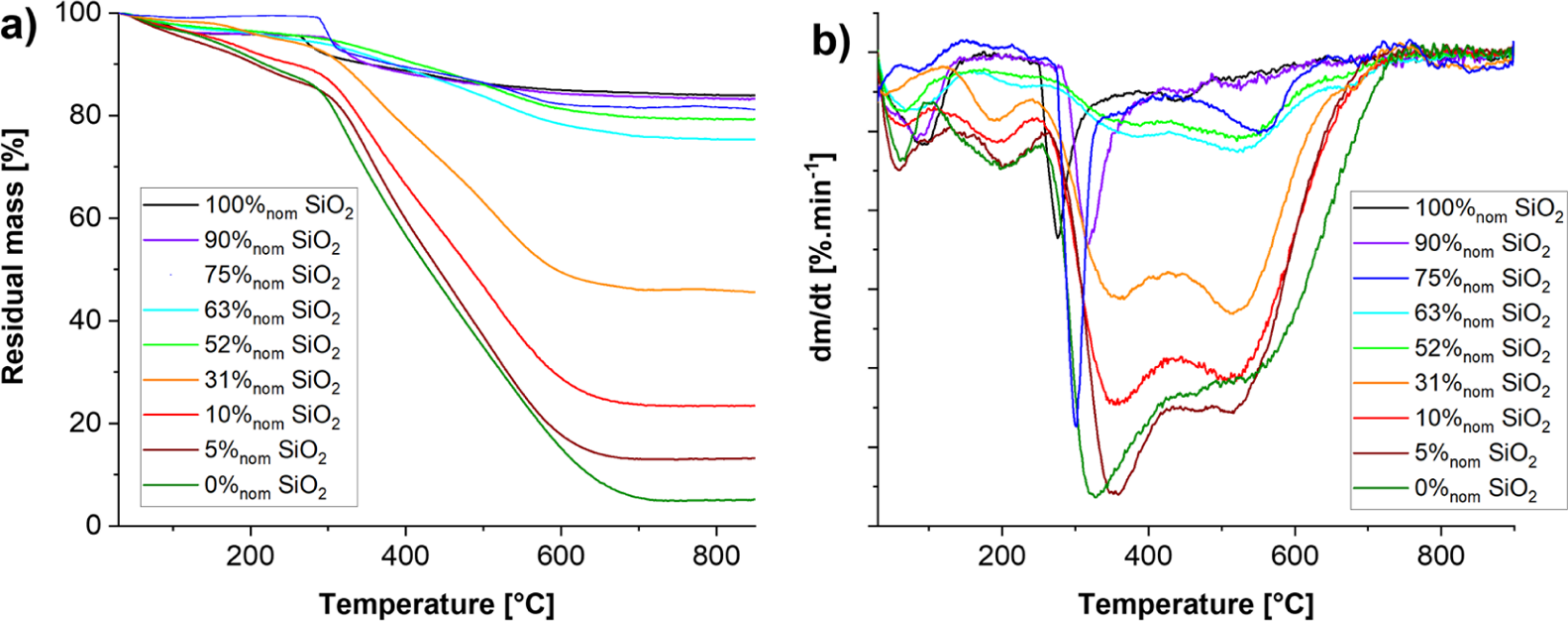
TGA curves of silica aerogels, RMF resins, and their composites: (a) residual masses and (b) weight losses (Figure 2b).

TGA on a composite aerogel should decompose all its organic parts (resin phase and surface functional groups) under our measurement conditions (up to 800 °C in air), leaving behind a stoichiometric SiO_2_ residue, thus allowing for the determination of the true SiO_2_ content (Figure 2). In addition, the position of the weight loss peaks can give insights into the chemistry of the sample. For silica aerogels synthesized from TEOS, some surface ethoxy groups can be hydrolyzed, depending on the chemical conditions during gelation, solvent exchange, and drying. This will result in different ratios of ethoxy and hydroxyl surface groups that lead to two peaks in the TGA weight loss curves at around 280 and 400 °C, respectively (Figure 2b). For composite samples, the decomposition of the surface groups partially overlaps with the two typical, broad weight loss peaks of RMF at ∼320 and ∼510 °C, making quantitative observations difficult. Nevertheless, the ethoxy weight loss peak clearly changes upon the addition of RMF: it shifts to higher temperatures and is no longer clearly distinguishable for silica contents ≤63%_nom_ SiO_2_. This indicates some change of the surface groups of silica with the addition of RMF.

To better constrain the chemical composition of the aerogels, C, H, and N concentrations were determined using elemental analysis. The oxygen composition in the organic part was estimated using the following formula
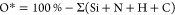


**Figure 3:**
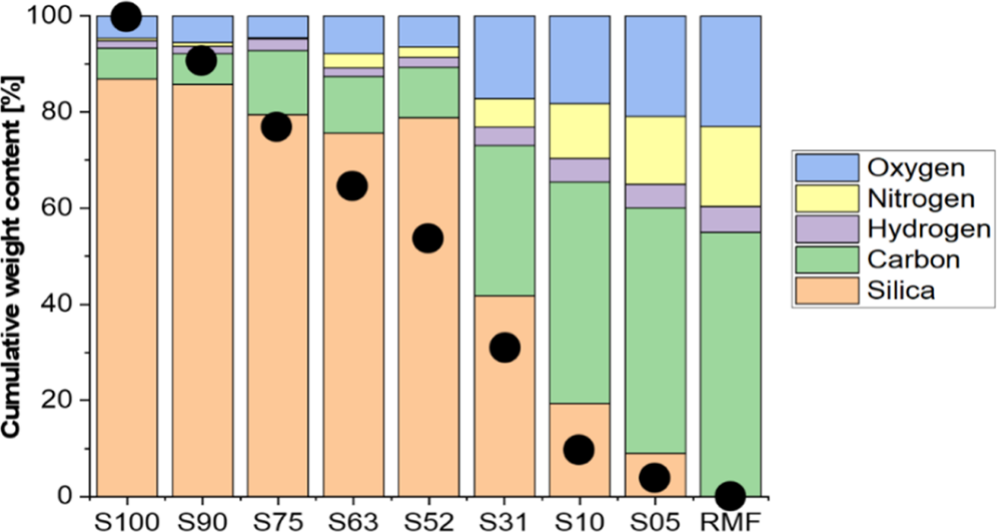
Estimated elemental composition of silica aerogels, RMF resins, and their composites. The black dots correspond to the nominal silica precursor contents.

The calculation was made with the silica content originating from TGA results and C, H, and N contents from elemental analysis, and the remaining mass was attributed to oxygen atoms that were not bound to silica—originating from the RMF structure and silica-bound ethoxy and hydroxyl surface groups (Figure 3).

Figure 3 compares the measured composition to their nominal composition. Due to the similar molecular mass of SiO_2_ (*M* = 60.08 g·mol^–1^) and R + M + F (*M* = 57.46 g·mol^–1^), the molecular (%_nom_) and weight (%_wt,nom_) nominal composition is nearly equivalent. For compositions >90%_nom_ SiO_2_, the measured silica content is lower than the nominal one. This is simply due to the presence of the surface groups on the large specific surface of the silica aerogel (ethoxy and hydroxyl). If these surface groups would remain unchanged upon the addition of RMF, which does not seem to be the case based on the observed changes in the ethoxy weight loss peak, one could thus expect a systematic underestimation of the organic content in the calculated nominal composition. If, on the other hand, the surface groups are partially or fully replaced by the RMF reactants, this will lead to an increased RMF content without changing the measured silica content. There are also factors leading to an overestimation of the silica composition. Namely, for the composites, we attributed all remaining mass after TGA up to 800 °C to silica. However, the pure RMF also showed a residue of 5%_wt_ which clearly cannot be attributed to any specific phase. Thus, the SiO_2_ content will be slightly over- and the oxygen content underestimated for the composite samples. Another factor leading to a larger nominal silica content is the water formed during condensation of R + M + F species into an RMF resin, lowering the weight of the resin compared to the reactants. Indeed, for composites with ≤75%_nom_ SiO_2_, the measured silica content is higher than the nominal one. Although the factors discussed above certainly contribute to this difference between nominal and measured compositions, there are two factors which indicate that at least for samples with 75 and 52%_nom_ SiO_2_, the RMF yield is less than 100%, and some of the RMF reactants get partially washed away during solvent exchange: first, for the 75%_nom_ SiO_2_ sample, no significant nitrogen concentration is observed, which is contrary to expectations based on the nominal composition. Second, for compositions between 63 and 52%_nom_ SiO_2_, the measured silica content is almost constant or even increases with the decreasing nominal silica content. This is consistent with observations of particles in the exchange liquid (see the Supporting Information) and could arise from different degrees of integration for the RMF reagents in the materials’ backbone.

**Figure 4:**
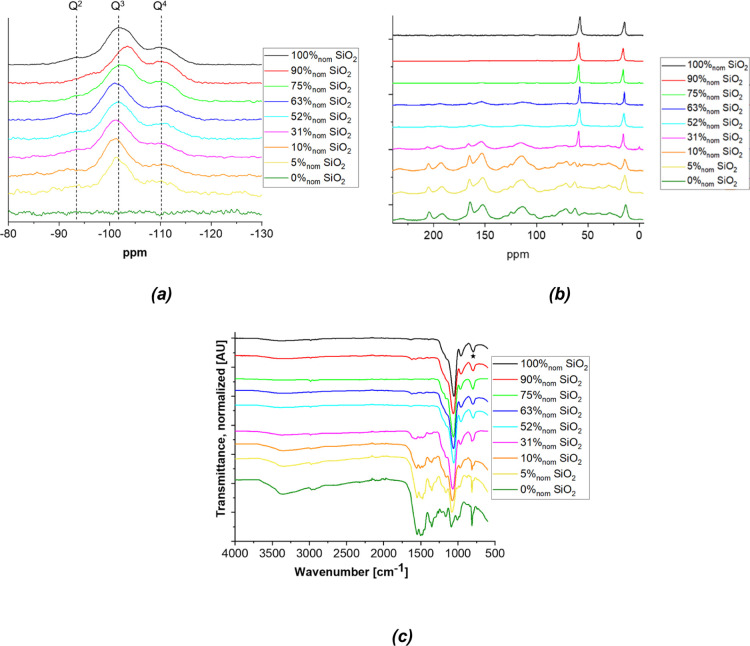
NMR and FTIR spectra of silica aerogel, RMF resin aerogel, and their composites. (a) ^1^H–^29^Si CP MAS NMR spectra, dotted lines correspond to the position with maximum intensity for the Q^2^, Q^3^, and Q^4^ peaks of the pure silica; (b) ^1^H–^13^C CP MAS NMR spectra; and (c) FTIR spectra, the star around 800 cm^–1^ indicates ethoxy groups.

To get further insights in the evolution of the bonding environment, ^1^H–^29^Si and ^1^H–^13^C NMR and FTIR spectra were acquired. All ^1^H–^29^Si NMR spectra (Figure 4a), except of course for the pure RMF material, display three silica peaks associated with Q^2^, Q^3^, and Q^4^ species where *n* indicates the number of bridging Si–O–Si oxygen atoms attached to the Si atom in question. The signal-to-noise ratio decreases with the decreasing silica content, despite partial compensation by increasing the amount of scans. The spectra are typical for silica aerogels: Q^2^ and Q^3^ species are predominantly present on the surface/interface, and Q^4^ makes up the bulk of the silica phase (ref Wim/Shanyu paper). Because of the cross polarization during the spectral acquisition, the peak intensities do not directly correspond to the Q^*n*^ abundances and can thus not be used directly for quantitative analysis: due to its inherently larger distance to ^1^H, Q^4^ abundance would be underestimated compared to Q^3^ and Q^2^. The position of the Q^3^ resonance gives further insights into changes in the bonding environment as it is sensitive to the nature of the non-bridging oxygen: Q^3^-OH has an expected peak position around 100 ppm, whereas Q^3^-O–C typically has a resonance around 104 ppm, regardless of the bonding environment of the carbon.^57−59^ The pure silica aerogel (S100, 100%_nom_ SiO_2_) has a relatively broad Q^3^ peak near 102 ppm indicating a mixture of silanol and ethoxy groups on the non-bridging oxygen. Upon the addition of small amounts of RMF (75–90%_nom_ SiO_2_, with a lower real RMF content), the Q^3^ peak shifts to more negative ppm values, indicating additional organic groups (−Si–O–C−) chemically bound to the silica. Further addition of RMF, on the other hand (≤63%_nom_ SiO_2_), leads to a shift in the opposite direction toward less negative ppm values, indicating more silanol surface groups than in the pure silica. A possible exception is the sample with 52%_nom_ SiO_2_, where the peak seems to be similar to the one of the pure silica. However, this is also the sample with the largest discrepancy between the nominal and measured silica content. According to the measured silica content, the sample should be in-between those with 75 and 63%_nom_ SiO_2_, which would make it consistent with the trend described above.

The reasons for the observed changes are likely different gelation and integration mechanisms of the RMF phase. At a low RMF content (75–90%_nom_ SiO_2_), a significant amount of the additional organic groups seems to react with the surface of the silica phase. At higher RMF concentrations, the degree of interaction seems to increase, and even more Si–OH are observed than in pure silica. There are different possible explanations for this. The organic groups could attach themselves to the silica surface at an early stage of the gelation process, partially replacing ethoxy groups from the surface. At higher RMF concentrations, however, this step could be followed by a detachment and replacement of the originally attached organic groups by hydroxyl groups. This could, for example, be driven by the nucleation and growth of a separate resin phase (see TEM data below). Alternatively, the increase in Si–OH at higher RMF contents could simply be related to a shift in the hydrolysis–alcoholysis equilibrium due to the higher water content in the RMF sol—however, in this case, we would expect a more gradual and continuous change with an increasing nominal RMF content.

The ^1^H–^13^C NMR and FTIR spectra reveal less details and similar general information. Measured spectra for the neat silica and neat RMF materials are generally as expected for those systems. For both methods, the silica-related peaks are more narrow and better defined compared to the much broader RMF-related peaks, as expected for carbons in ethoxy groups *versus* carbons that are part of RMF resin (with very large chemical shift anisotropy). This means that even for the 31%_nom_ SiO_2_ sample, which has a measured silica content of only 42%_wt_, the spectra seem to be dominated by the SiO_2_-related peaks. Neither of the methods can be used for quantitative analysis; NMR due to the cross-polarization discussed for the ^1^H–^29^Si spectra and FTIR due to the ATR-based acquisition conditions and due to the high variability in molar extinction coefficients. However, for both techniques, the spectra change gradually from a typical silica spectrum to the one of RMF with a decreasing measured silica content. Interestingly though, the signal coming from the ethoxy groups does not vanish for either NMR or FTIR, even for the pure RMF. The ^1^H–^13^C NMR of the pure RMF aerogel (Figure 4b), although similar to the one of a classical water-based RMF recipe,^39^ differs from it by the presence of an additional peak around δ = 14 ppm. This peak likely can be assigned to the terminal carbon of an ethoxy group, possibly arising through the etherification of some of the hydroxymethyl derivatives formed during the initial stages of synthesis. Similarly, the FTIR spectra of pure RMF display the typical RMF peaks in the 1300–1700 cm^–1^ region, thoroughly described elsewhere.^39^ However, there is a shoulder present around 800 cm^–1^ (indicated by a star symbol on the graph) which can again be attributed to unhydrolyzed ethoxy groups and an increased intensity in the 1100 cm^–1^ region. The assumption of the presence of ethoxy groups in the RMF phase is based on a similar mechanism described elsewhere for other alcoholic systems with aromatic molecules, including melamine^60^ and hydroxymethylfurfural,^61^ similar to the esterification mechanism also observed in silica, with the replacement of terminal O–H groups by surrounding R-OH alcohols.^62^ This means that care has to be taken not to restrict the interpretation of the presence of ethoxy groups as forcibly being part of the silica surface.

### Microstructure

**Figure 5:**
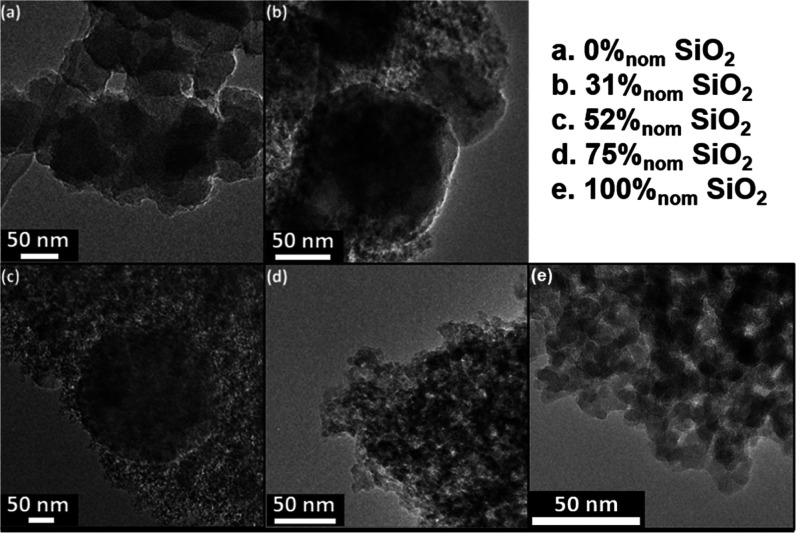
TEM micrographs of RMF–silica aerogels with a gradually increasing nominal silica content. Note the difference in magnification for different images; scale bars have been adjusted to represent 50 nm in all images. (a) Corresponds to the pure RMF resin, (b–d) corresponds to silica–RMF composite aerogels, and (e) corresponds to the pure silica aerogel.

TEM imaging shows the expected colloidal nature and random aggregation behavior for both the pure silica and the pure RMF samples; however, the size of primary particles forming the agglomerates is very different for silica (∼5–10 nm diameter, Figure 5e) and RMF (∼80 nm diameter, Figure 5a). For composites with only small amounts of RMF, no separate RMF phase can be observed (75%_nom_ SiO_2_, Figure 5d). For larger RMF concentrations (52 and 31%_nom_ SiO_2_, Figure 5b–c), distinct RMF particles can be observed. However, while the size of the silica primary particles remains similar, the size of the RMF particles in the composite systems is significantly larger (200–400 nm) than in the pure RMF sample. There are two different possible explanations for the observed increase in the RMF primary particle diameter: either the presence of the silica slows down the nucleation of the primary particles compared to their growth, leading to larger particles, or the morphological changes originate from variations in the water/ethanol ratio between recipes, arising from the different precursors added during synthesis. Another interesting point is that while the silica phase seems to be continuous for all composites observed *via* TEM, the RMF particles in the 52 and 31%_nom_ SiO_2_ systems seem to be well integrated in the silica phase but isolated from each other.

In the absence of specific interactions and effects, the envelope and skeletal densities of a physical mixture resemble the volumetric average between its components. To be able to estimate the properties of such a mixture, we need to know the envelope and skeletal density of the pure RMF and SiO_2_ phases. For the RMF/organic phase (ρ^org^), we can use the measured properties of the pure RMF. For silica, the situation is more complex, as even the 100%_nom_ SiO_2_ aerogel contains a significant amount of organics due to the presence of surface groups. However, for any composite with a silica content of , assuming that the density of the organic phase remains constant, we can estimate the skeletal density of the silica from eq 3.
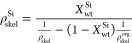
3

On applying this formula to the two samples with >85%_wt_ SiO_2_, the skeletal density of the pure silica phase was estimated to be 1.77 ± 0.01 g/cm^3^. To estimate the envelope density of the pure silica phase, a slightly different approach was used. The disparity between the porosity in the organic and in the silica phase being very large, we can neglect any contribution of the organic phase to the porosity in the 100%_nom_ SiO_2_. Thus, the envelope density of the silica phase can be estimated from the results of the 100%_nom_ SiO_2_ sample, using the total pore volume to solid volume ratio, that is, porosity, , according to eq 4.

With the definitions of pore volume, , and pore fraction, 
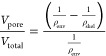
we have
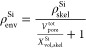
4

Once both the skeletal and envelope (ph ∈ [skel, env]) densities of the pure phases are known, the expected total densities can be calculated from the weight percent of silica (*X*_wt_^Si^) using eqs 5 and 6.
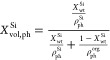
5
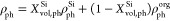
6

**Figure 6:**
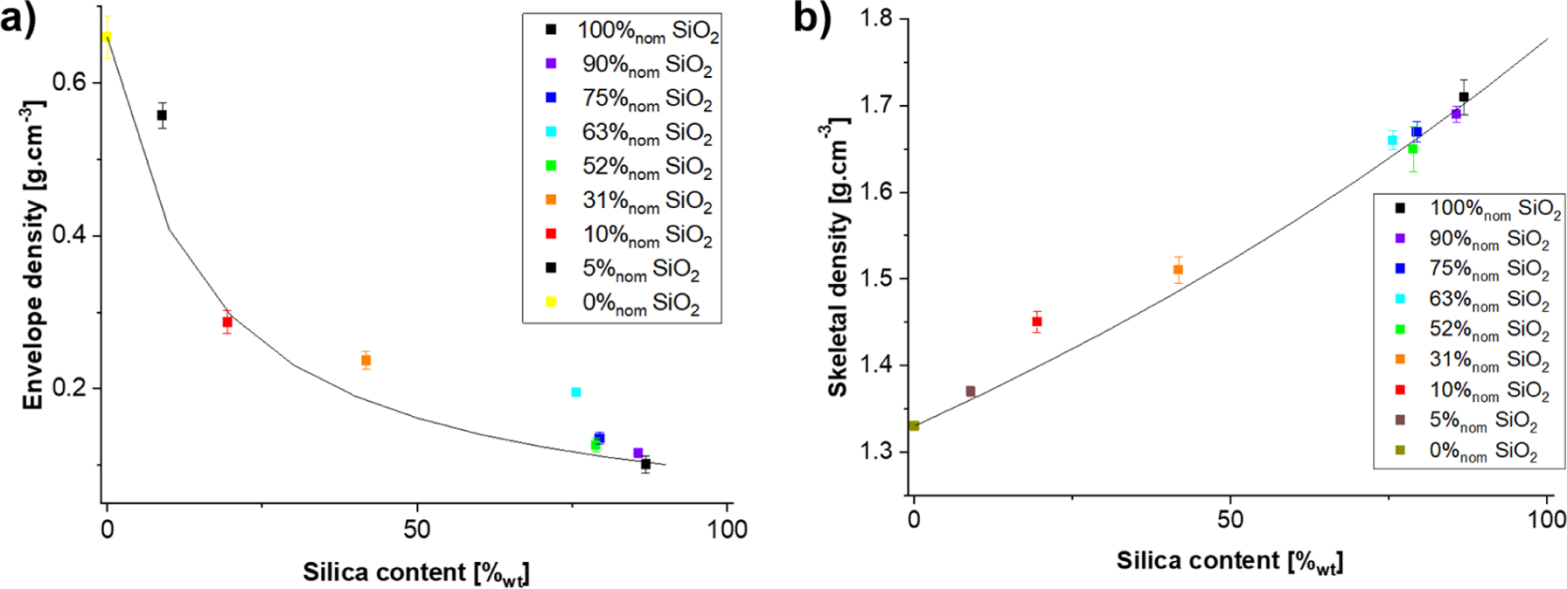
Envelope density (a) and skeletal density (b) of silica aerogels, RMF resins, and their composites. The black lines correspond to the expected volumetric average over the two phases.

Figure 6 shows both the measured densities and the trends according to eq 6. It can be seen that while the skeletal densities largely follow the expected trend, the envelope densities of the composites seem to have a tendency of larger than expected densities.

To get further insights into this possible densification, the porosity of the samples has been assessed using nitrogen sorption analysis, which probes the porosity in the scale of ∼1–50 nm. Nitrogen physisorption usually results in a calculation of the surface area/pore volume per gram of material. Although this assessment is adapted to single-component systems, composites have different skeletal densities: a given volume of an RMF solid is lighter than the same volume of amorphous silica, leading to a large volumetric variation in what constitutes a gram of solid material. Therefore, surface areas and mesopore volumes are instead discussed in, respectively, m^2^/cm^3^ and cm^3^/cm^3^, where the volume in the denominator is the skeletal volume (1/ρ_skel_).

**Figure 7:**
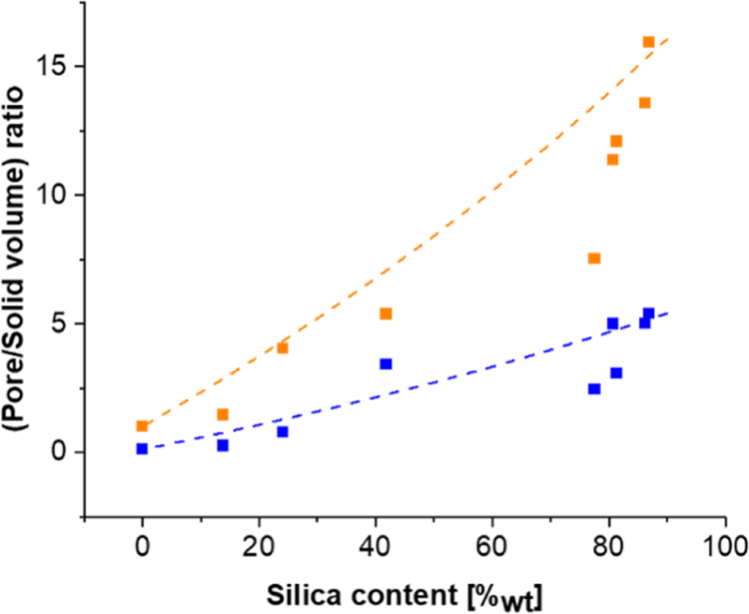
Evolution of the total (orange symbols) and NLDFT (blue symbols) pore to solid volume ratio for different composites and the expected value for volumetric mixtures (dotted lines).

The porosity assessment of silica–RMF composites is crucial because while silica aerogels have a high fraction of mesoporosity, RMF resins are mostly macroporous. Thus, the porosity of their composites is expected to lie somewhere in between, with the exact values being dependent on how the two phases connect with each other. Indeed, the surface area *S* of the samples gradually decreases from typical values for silica aerogels (1428 m^2^/cm^3^ or 835 m^2^/g for *S*_NLDFT_ and 1573 m^2^/cm^3^ or 920 m^2^/g for *S*_BET_) to typical values for RMF resins (77 m^2^/cm^3^ or 67 m^2^/g *S*_NLDFT_ and 100 m^2^/cm^3^ or 51 m^2^/g *S*_BET_) with a decreasing silica content (see the Supporting Information for the full data set). To understand a possible densification, we compared both the measured NLDFT pore to solid volume ratio (*V*_pore/sol_^NLDFT^) and the total pore to solid volume (*V*_pore/sol_^tot^) to the expected trends (*V*_pore/sol_ = *X*_vol,skel_^Si^*V*_pore/sol_^Si^ + (1 – *X*_vol,skel_^Si^)*V*_pore/sol_^org^). The results (Figure 7a) indicate that the deviation between the experimental data and predicted trend, that is, the additional densification, happens mainly at the larger-scale porosity (≥50 nm) rather than at the smaller-scale porosity as assessed with NLDFT. Finally, the characteristic size of the porosity (*d* = *V*_pore/sol_^NLDFT^/*S*^NLDFT^) has been calculated (Figure 7b). Changes in the characteristic size can indicate either a change in shape (changing the geometric pre-factor which has not been taken into account here) or a change in the typical size of the porosity. There is no clear general trend with the silica content discernible, but two observations might be of note: while neat RMF has a significantly smaller characteristic size than silica, the characteristic size of the composites is closer to the one of silica than the one of RMF. This is consistent with the fact that the NLDFT pore volume contained in the silica phase is significantly larger than the one of the RMF. Of course, the size of all pores is still likely to be larger in RMF than in silica, as only about 12% of the pore volume is probed by the nitrogen sorption, while in silica, it is above 33%. Second, it is interesting to look at the samples with ≥52%_nom_ SiO_2_. Here, different trends can be observed: while for the composites with 75 and 90%_nom_ SiO_2_, the characteristic size seems to increase, the inverse can be observed for the 52 and 63%_nom_ SiO_2_ materials, despite the fact that the measured SiO_2_ content in the 75 and 63%_nom_ SiO_2_ samples is almost the same. This strengthens our previous observations that the RMF integration mechanism for samples ≥75%_nom_ SiO_2_ is different from that of the ones with 63%_nom_ SiO_2_.

Together with the chemical identity assessment, the microstructural analysis allows us to formulate a hypothesis with some confidence on what happens in these composite systems during gelation. At high nominal silica concentrations (≥75%_nom_ SiO_2_), organic species from the RMF precursors seem to partially replace the hydroxyl and ethoxy groups from the silica surface, leading to a shift in the ethoxy peak observed in TGA and a shift of the Q^3^ NMR peak indicating more carbon-rich environments. The additional organic surface groups seem to contain little to no nitrogen, indicating that melamine might contribute less to their formation and is likely to be washed out partially during solvent exchange. The interaction between the RMF precursors and silica surfaces, together with the lower initial RMF concentration in the sol, seems to prevent the formation of a separate RMF-resin phase: no such phase can be observed in TEM. It is likely that an initial adsorption of RMF precursors on the silica surfaces present in the sol happens even in systems with a lower silica content. However, for systems ≤63%_nom_ SiO_2_, RMF particles do nucleate and grow as can be seen from the TEM images. However, both the dilution of the RMF with the addition of the silica sol and the interaction between RMF precursors and silica surfaces lead to a lower super-saturation with respect to the resin compared to the pure sol. This results in much larger primary RMF particles and will likely also slow down the nucleation and growth kinetics. At least for systems between 63 and 52%_nom_ SiO_2_, the formed RMF particles seem to be only partially integrated in the aerogel, either due to their size leading to precipitation/segregation or due to the slow nucleation/growth kinetics. With even a lower silica content, the RMF particles seem to be well integrated in the silica phase. The formation of RMF particles seems to lead to the at least partial desorption of precursor species from the silica surface and their integration into the RMF phase. These surface groups seem to be replaced by hydroxyls, leading to another shift of the NMR Q^3^ peak.

### Mechanical Properties

The mechanical compression behavior of aerogels is complex and not fully understood. For our composites, similar to previous observations on silica aerogels,^63^ a viscoelastic plateau with a characteristic minimum modulus is observed, followed by a region with an increasing modulus and the appearance of localized failure and finally global failure after a maximum stress is reached (see the Supporting Information). Some samples displayed clear signs of macroscopic defects during the measurements—these measurements were not used for further analysis. For the remaining measurements, three different quantities were determined: the minimum of the modulus in the viscoelastic plateau, the maximum stress reached and the strain at maximum stress, and the stress and strain at the first clearly discernible localized failure. Although the definition of the first two is clear, the first localized failure is less clear. We used two criteria for the definition: the modulus needed to drop locally below zero, and a discontinuity of the stress–strain curve needed to be visible.

**Figure 8:**
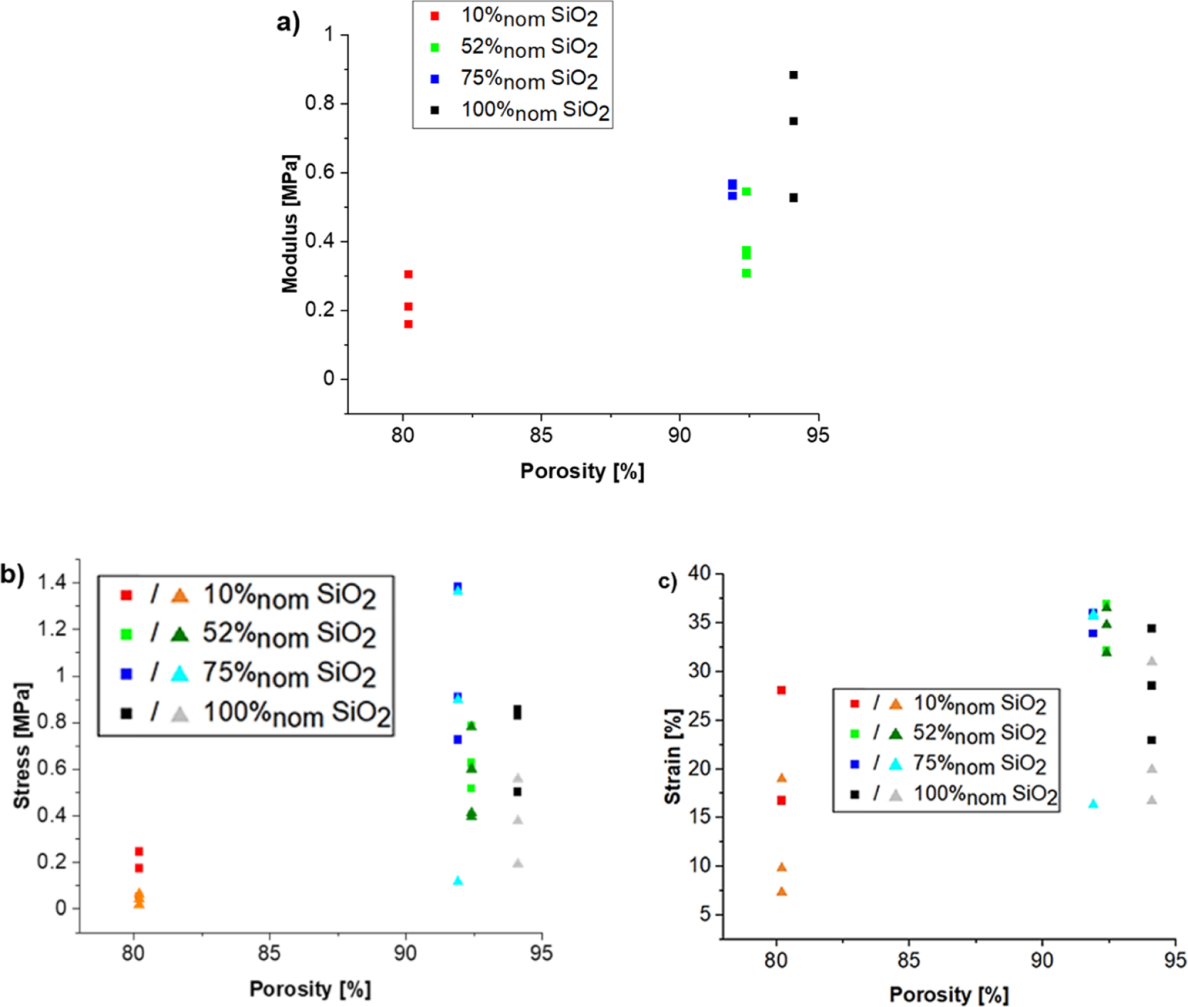
Compressive properties of silica–RMF composite aerogels as a function of porosity. (a) *E* modulus from the quasilinear portion of the compression test (*i.e.*, low strains), (b) stress at failure (triangles) and maximum stress (squares), and (c) strain at failure (triangles) and maximum strain (squares).

The compression of silica aerogels reveals various behaviors dependent on the actual silica concentration and the microstructural distribution of the two phases (Figure 8). Compared to the neat SiO_2_ sample, the samples with 75%_nom_ SiO_2_ (with organic groups at the silica surfaces) seem to show a moderate improvement of the mechanical properties. Although the minimum modulus of the viscoelastic plateau is in the lower range of what has been measured for the pure system, the samples support both a higher stress and a higher strain before both localized and global failures. For lower amounts of silica, resulting in samples with RMF particles embedded within the silica phase, no significant improvement of the mechanical properties can be found. At 52%_nom_ SiO_2_, resulting in a sample with a similar measured SiO_2_ content as the 75%_nom_ SiO_2_, the minimal modulus is lower than the one of the pure silica sample, and while a similar stress is reached before failure, the resulting strain is larger indicating an overall lower modulus. Finally, samples with the least amount of silica (10%_nom_ SiO_2_) are overall significantly weaker, failure occurs at lower stresses, and a lower minimal modulus than that of pure silica aerogel is observed. This is likely a result of the lack of connectivity of the RMF phase. Indeed, the incorporation of large RMF particles which are much less compressible than those of the silica phase is likely to lead to strain concentrations in the silica phase. Additionally, the mechanical property of the silica–RMF interface is not well known and could contribute to a weakening of the composites. In conclusion, while the adsorption of additional groups at the surface of the silica seems to lead to a moderate improvement of the mechanical properties, the inclusion of large, isolated RMF particles in the silicate phase, while having little effect on the thermal conductivity (see below), does not improve the mechanical properties, and depending on the concentration can even have a detrimental effect.

### Thermal Conductivity

To estimate the performance of the composites for different applications, their thermal conductivity and mechanical compression behavior was measured. For the thermal conductivity, we again compare to trends for mechanical mixtures. For this, we estimated the two limiting cases of purely parallel and purely serial resistances from the two phases. As for the densities, we first needed to estimate the thermal conductivity of the pure silicate phase in the serial (eq 7) and parallel case (eq 8) from the thermal conductivity measured for the 100%_nom_ SiO_2_ sample.
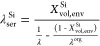
7
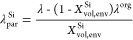
8

Once the conductivity of the pure silica phase is calculated, we can estimate the expected thermal conductivities for the serial and parallel case with eqs 9 and 10, respectively.
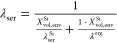
9
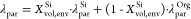
10

**Figure 9:**
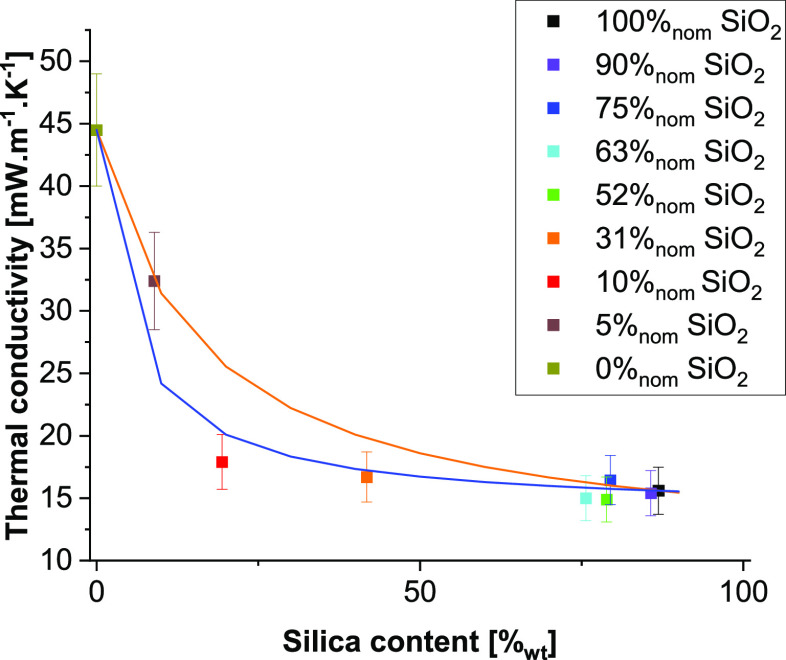
Thermal conductivity of silica aerogels, RMF resins, and their composites. Also shown are the two limits for parallel (orange) and serial (blue) resistances.

The results of the thermal conductivity are shown in Figure 9. The thermal conductivity of silica–RMF composites (Figure 9) with high silica contents (≥52%_nom_ SiO_2_) displays variations that are all within the measurement accuracy, allowing no conclusions with respect to differences in behavior. For an intermediate silica content, (10–31%_nom_ SiO_2_) thermal conductivity clearly follows the trend of serial resistances, while for a lower silica content (5%_nom_ SiO_2_), the measured thermal conductivity is closer to that of the parallel case. This is consistent with the observed lack of interconnectivity between the RMF particles down to silica contents as low as 10%_nom_ SiO_2_. The measured thermal conductivity of the 5%_nom_ SiO_2_ sample, on the other hand, indicates a continuous RMF network having formed throughout the aerogel volume. These results indicate a threshold for high insulation performance somewhere between 5 and 10%_nom_ SiO_2_, corresponding to 14–24%_wt_ and 50–70%_vol_ of SiO_2_, this threshold being due to a progressive connectivity of RMF particles happening throughout the material.

## Conclusions

In this work, we have taken advantage of the compatibility of two sol–gel systems—TEOS-based silica and RMF alcogels—to produce RMF–silica hybrid aerogels by supercritical drying of the alcogels. Thorough chemical and microstructural analysis and measurement of the resulting properties have provided an in-depth understanding of the gelation process and resulting microstructure within the composites. At a high silica content (≥75%_nom_ SiO_2_), the organic phase gets incorporated as additional surface groups or even a surface coating of the silica phase. Contrary to the precursors, this surface coating contains little to no nitrogen. At lower silica contents, a separate RMF resin phase forms. For silica contents ≃75%_nom_ SiO_2_, the RMF phase is integrated as large (200–400 nm), isolated particles within the silica phase. Either due to the interaction of the RMF precursors with the silica phase or due to dilution, the size of these particles is significantly larger than that of the primary particles in the pure RMF resins. At intermediate silica levels (52–63%_nom_ SiO_2_), the RMF particles are only partially integrated in the aerogels, leading to final compositions that differ from their (expected) nominal composition. At silica contents ≤5%_nom_ SiO_2_, a connectivity of the RMF phase is reached, significantly changing the thermal conductivity which, up to this point, remains similar to that of pure silica aerogels. Although the addition of an organic surface coating leads to a moderate improvement of the mechanical properties, the isolated RMF particles have no such effect and, depending on the concentration, can even be detrimental. With their tailorable microstructure and very low thermal conductivity, RMF–silica composite aerogels have potential applications for thermal insulation or as green bodies for the production of silica–carbon composite aerogels.

## Supporting Information Available

Appearance of first solvent exchanges from silica–RMF composite alcogels after gelation, for visual inspection; full numerical values from elemental analysis; numerical values from the porosity assessment of silica–RMF composite aerogels arising from nitrogen physisorption, helium pycnometry, and sand displacement; numerical values from the porosity assessment of aerogels, normalized by volume of the solid phase rather than mass; skeletal density of silica–RMF composite aerogels and its expected trend based on the nominal silica content; thermal conductivity of silica–RMF aerogels and its expected trend based on the nominal silica content; curve arising from a compression test realized on a silica–RMF composite aerogel and its analysis regarding local and global mechanical failures; and compressive mechanical tests realized on various compositions of silica–RMF composite aerogels (PDF)
