# Supplementary material for: Silica–Resorcinol–Melamine–Formaldehyde Composite Aerogels as High-Performance Thermal Insulators
Source: ACS Omega. 2022 Apr 21;7(17):14478–89. doi: 10.1021/acsomega.1c04462 (PMC9088793; doi:10.1021/acsomega.1c04462)
Supplement: Supplementary file 1 — ao1c04462_si_001.pdf [file ao1c04462_si_001.pdf]

Supplementary Information

Silica – Resorcinol-Melamine-Formaldehyde composite aerogels as high performance thermal insulators

Romain Civioc [1,2*], Wim J. Malfait [1], Marco Lattuada [2], Matthias M. Koebel [1], Sandra Galmarini [1*]

[1] Laboratory for Building Energy Materials and Components, Swiss Federal Laboratories for Materials Science and Technology, Empa, Duebendorf

[2] Department of Chemistry, University of Fribourg, Chemin du Musée 9, 1700 Fribourg, Switzerland

** corresponding authors: romain.civioc@empa.ch, sandra.galmarini@empa.ch*

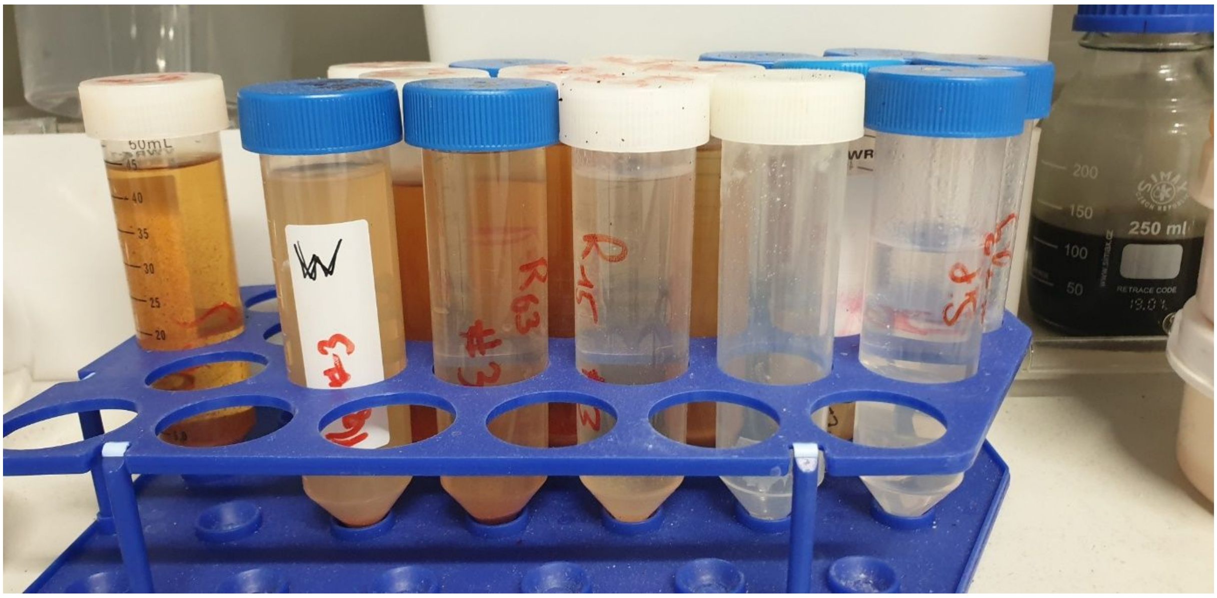

Figure S1: Appearance of first solvent exchanges from silica-RMF composite alcogels after gelation, with the initial RMF content decreasing from left to right.

Name	C wt%	H wt%	N wt%	SiO_2 wt%	Organic O wt%
**S100**	6.36	1.64	0.51	86.88	4.63
**S90**	6.47	1.58	0.67	86.20	5.09
**S75**	13.38	2.45	0.24	79.43	4.51
**S63**	11.73	1.82	2.96	77.55	5.95
**S52**	10.50	2.01	2.25	81.31	3.95
**S31**	31.26	3.82	5.91	41.77	17.24
**S10**	46.13	4.78	11.39	24.03	13.67
**S05**	51.20	4.81	14.17	13.80	16.02
**RMF**	55.02	5.30	16.64	0	17.92

Table S1: Elemental composition numerical values used for Figure 2 of the main text.

Table S2: Porosity assessment of silica aerogels, RMF resins and their composites by nitrogen physisorption, helium pycnometry, and sand displacement. Results are displayed in $m^2.g^{-1}$ for surfaces and $cm^3.g^{-1}$ for volumes.

Sample Name	Silica content [$\%_{wt}$]	Surface Area BET-Rouquerol [$m^2.g^{-1}$]	Surface Area NLDFT [$m^2.g^{-1}$]	Pore Volume NLDFT [$cm^3.g^{-1}$]	Pore Volume from densities [$cm^3.g^{-1}$]
S100	86.9	920	835	3.16	9.32
S90	85.7	931	693	2.96	8.03
S75	79.4	1009	668	2.99	6.81
S63	75.6	695	678	1.47	4.53
S52	78.9	784	585	1.87	7.33
S31	41.8	970	594	2.28	3.56
S10	19.4	191	139	0.55	2.79
S05	8.9	73	56	0.19	1.07
RMF	0	51	67	0.09	0.76

Table S3: Porosity assessment of silica aerogels, RMF resins and their composites by nitrogen physisorption, helium pycnometry, and sand displacement. Due to changing skeletal densities, values have been normalized by volume of the solid phase rather than mass, making the values comparable between samples.

**Silica nominal content [$\%_{nom}$]**	**BET Surface [$m^2.cm^{-3}$]**	**NLDFT Surface [$m^2.cm^{-3}$]**	**NLDFT Pore Volume [$cm^3.cm^{-3}$]**	**Total Pore Volume [$cm^3.cm^{-3}$]**
100%	1573	1428	5.40	15.94
90%	1573	1171	5.00	13.57
75%	1685	1116	4.99	11.37
63%	1154	1125	2.44	7.52
52%	1294	965	3.09	12.09
31%	1465	897	3.44	5.38
10%	277	202	0.80	4.05
5%	100	77	0.26	1.47
0%	68	89	0.12	1.01

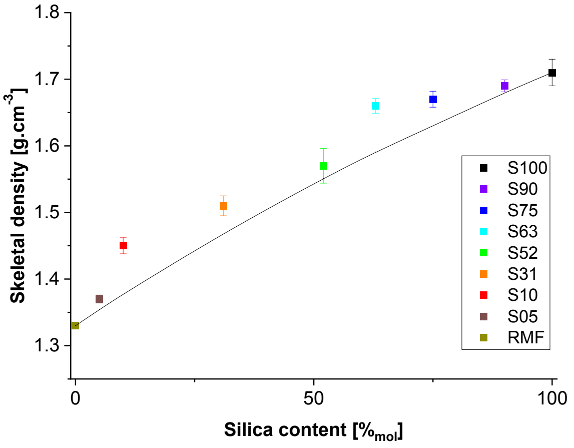

Figure S2: Skeletal density of silica/RMF composites and the expected trend (line) using their nominal silica content as opposed to the effective contents as found in the main text of the publication.

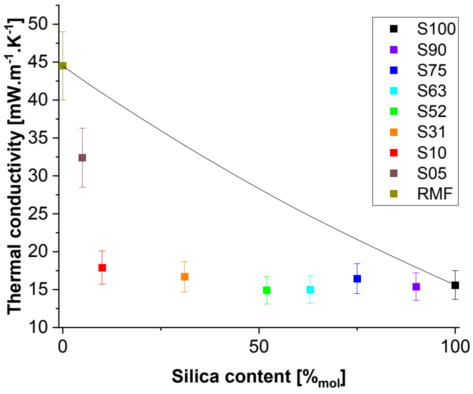

Figure S3: Thermal conductivity of silica/RMF composites and the expected trend (line) using their nominal silica content as opposed to the effective contents as found in the main text of the publication.

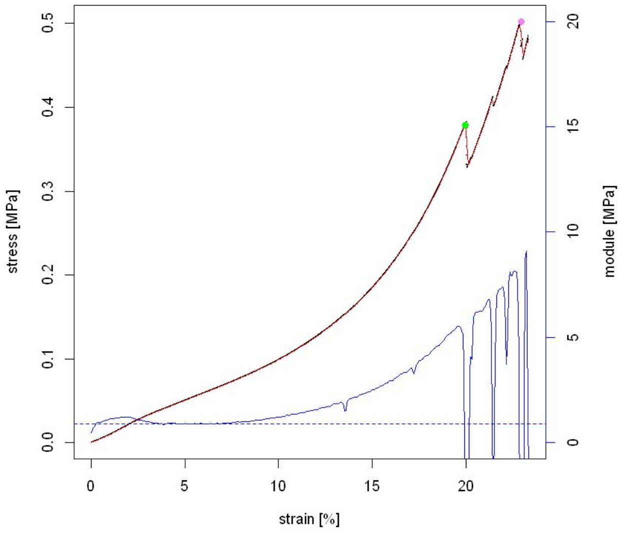

Figure S4: Typical appearance of a compressive mechanical test realized on a silica-RMF aerogel (red) and its corresponding derivative curve (blue), used to calculate the modulus and establish local failures due to buckling (indents on the derivative curve) as opposed to proper mechanical failure (large dips on the derivative curve).

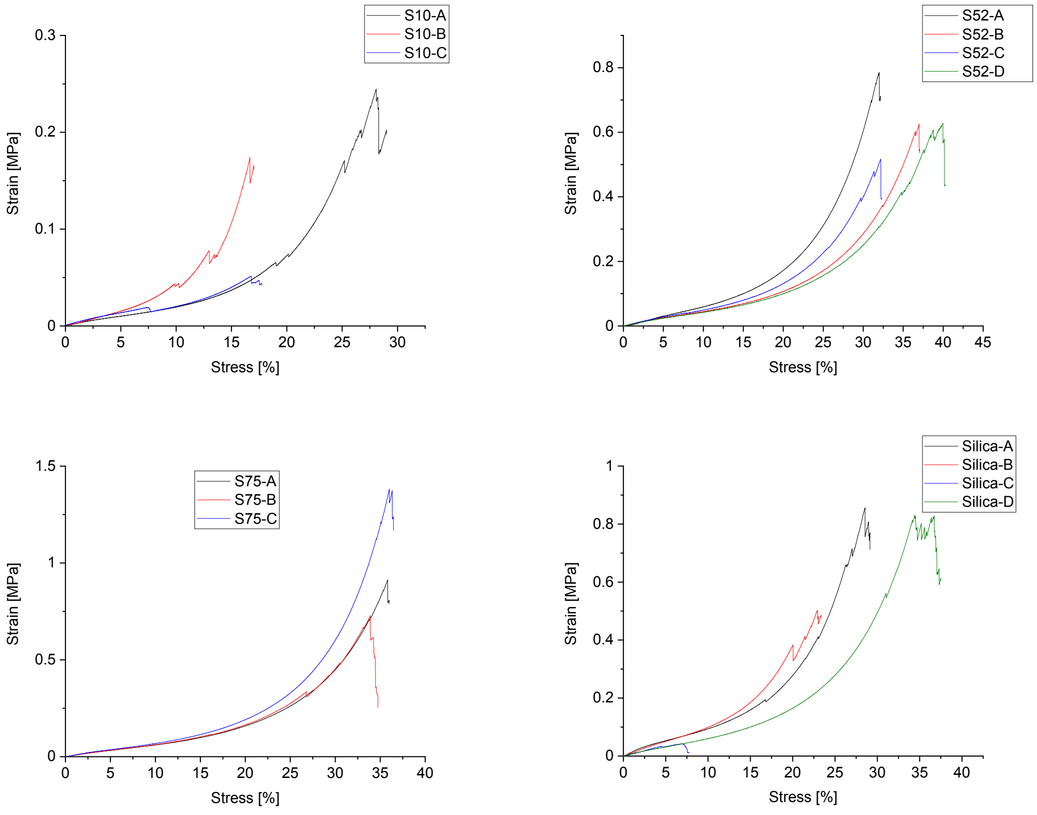

Figure S5: Compressive mechanical tests realized on different compositions of silica-RMF composite aerogels, as well as neat silica aerogels.
